# *Criblamydia sequanensis* Harbors a Megaplasmid Encoding Arsenite Resistance

**DOI:** 10.1128/genomeA.00949-14

**Published:** 2014-10-23

**Authors:** Claire Bertelli, Alexander Goesmann, Gilbert Greub

**Affiliations:** a Center for Research on Intracellular Bacteria, Institute of Microbiology, University Hospital Center and University of Lausanne, Lausanne, Switzerland; b SIB Swiss Institute of Bioinformatics, Lausanne, Switzerland; c University of Giessen, Systembiologie, Giessen, Germany

## Abstract

*Criblamydia sequanensis* is an amoeba-resisting bacterium recently isolated from the Seine River. This *Chlamydia-*related bacterium harbors a genome of approximately 3 Mbp and a megaplasmid of 89,525 bp. The plasmid encodes several efflux systems and an operon for arsenite resistance. This first genome sequence within the *Criblamydiaceae* family enlarges our view on the evolution and the ecology of this important bacterial clade largely understudied so far.

## GENOME ANNOUNCEMENT

For a few years, new *Chlamydia-*related bacteria are regularly reported in samples from various environments and animals, supporting a large biodiversity within the *Chlamydiales* order. In 2006, *Criblamydia sequanensis* was recovered from a water sample of the Seine River and isolated using amoebal coculture with the ubiquitous *Acanthamoeba castellanii* (1). It is the type strain of the *Criblamydiaceae* family (2) that was lately broadened by the report of a new species called *Estrella lausannensis* (3). The latter was isolated from raw surface water samples taken from a Spanish water-treatment plant (4). *C. sequanensis* presents a peculiar star-shaped elementary body on electron microscopy (1), suggesting a different cell-wall composition than other round *Chlamydiales* (5). Genome information is essential to broaden our understanding of this bacterial genus with an interesting structural phenotype.

The genomic DNA of *C. sequanensis* was sequenced on a GaIIx system (Illumina, San Diego) yielding 9,570,346 paired reads of 33 bp that were assembled with SOAPdenovo (6). Velvet (7) and Abyss (8) assemblies were used to solve some ambiguities within gaps by manual refinement, thus generating a draft assembly of 24 contigs larger than 1,000 bp organized in 21 scaffolds. The genome was automatically annotated by the GenDB system (9) with some manual improvement.

*C. sequanensis* presents one of the largest known chromosomes in the *Chlamydiales* order with a total contig size of 2,969,839 bp. The GC content is 38.2%. The chromosome draft is predicted to encode 2,426 genes. The bacterium likely possesses 4 ribosomal operons based on the coverage of contigs forming the ribosomal DNA. Moreover, this organism harbors a circular plasmid of 89,525 bp with a GC content of 40.8%, whose sequence has been completely solved.

The plasmid encodes 92 proteins, among which 26% do not have significant homologs in the NR database and 48% are conserved proteins of unknown function. Plasmid proteins seem to be of different origins, because they harbor the best BLAST hits against other *Chlamydiales* (25%), *Proteobacteria* (25%), *Bacteroidetes* (7%), *Firmicutes* (5%), *Cyanobacteria* (3%), *Planctomycetes* (1%) and *Archaea* (1%). In addition, 7% of the proteins are of phagic origin. The plasmid harbors several cation efflux systems that have orthologs in other *Chlamydia*-related bacteria like *Parachlamydia acanthamoebae, Protochlamydia amoebophila*, and *Simkania negevensis*. Among the 25% genes with a best hit against Proteobacteria is an unexpected operon for arsenite resistance that is not found in any other of the sequenced *Chlamydiales* but is commonly found in environmental bacteria (10). The detoxification capabilities are probably linked to particular conditions encountered by the bacteria in its natural environmental niche in the Seine River (11). The availability of a new plasmid sequence within the *Chlamydiales* order will help understanding the evolution of chlamydial plasmids, which were suggested by Collingro et al. (12) to originate from a conjugative megaplasmid acquired by a chlamydial ancestor.

### Nucleotide sequence accession numbers.

The whole-genome shotgun sequence of *Criblamydia sequanensis* strain CRIB-18 has been deposited in the European Nucleotide Archive under the accession numbers CCEJ010000001 to CCEJ010000023. The complete plasmid sequence of *C. sequanensis* strain CRIB-18 is available under accession number LK031773.
